# Supplementary material for: Optimizing the implementation of a participant-collected, mail-based SARS-CoV-2 serological survey in university-affiliated populations: lessons learned and practical guidance
Source: BMC Public Health. 2022 Oct 12;22:1907. doi: 10.1186/s12889-022-14234-1 (PMC9556138; doi:10.1186/s12889-022-14234-1)
Supplement: Supplementary file 1 — Additional file 1. Eligibility, Consent, and Demographic survey administered to UMass affiliates and their household members. REDCap surveys were administered to participants through campus email accounts. Supplemental Fig. 1. Instructional guides for collecting and repackaging samples. PDF versions of files were included with every box giving users instructions on how to collect blood spots and properly mail back the packages. [file 12889_2022_14234_MOESM1_ESM.pdf]

Instructions for Collecting your Dried Blood Spot (DBS) Sample

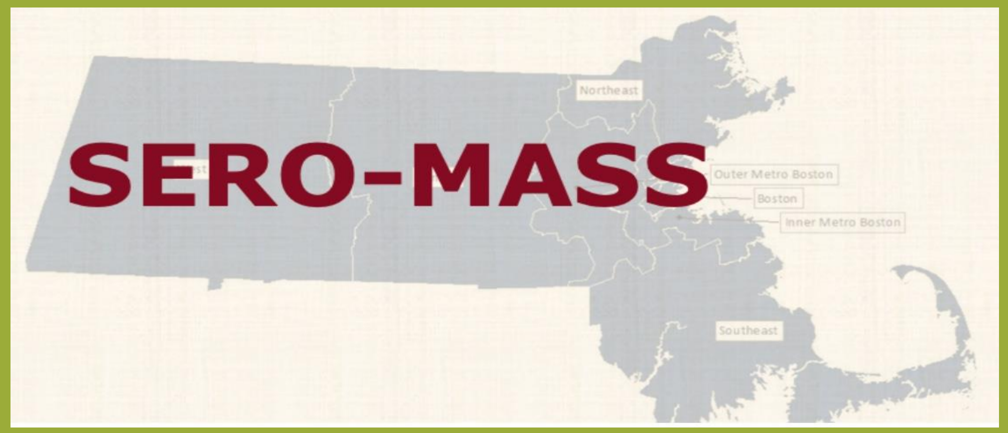

Conducted by:
The Lover Lab, Department of Biostatistics and Epidemiology, School of Public Health and Health Sciences, University of Massachusetts, Amherst
Lab Website: https://loverlab.io
&
The Alfandari Lab, Department of Veterinary and Animal Sciences (VASCI) & Developmental Biology, University of Massachusetts, Amherst

If you have any questions or concerns, please email:
covid19-antibody@umass.edu

If you need medical advice for this sample process,
please call the on-call nurse:
(413) 203-9836

Nurse Schedule:
Monday 10:00 am - noon
Wednesday & Friday 4:00 pm – 6:00 pm
Tuesday & Thursday 7:00 pm – 9:00 pm

DBS Collection Kit

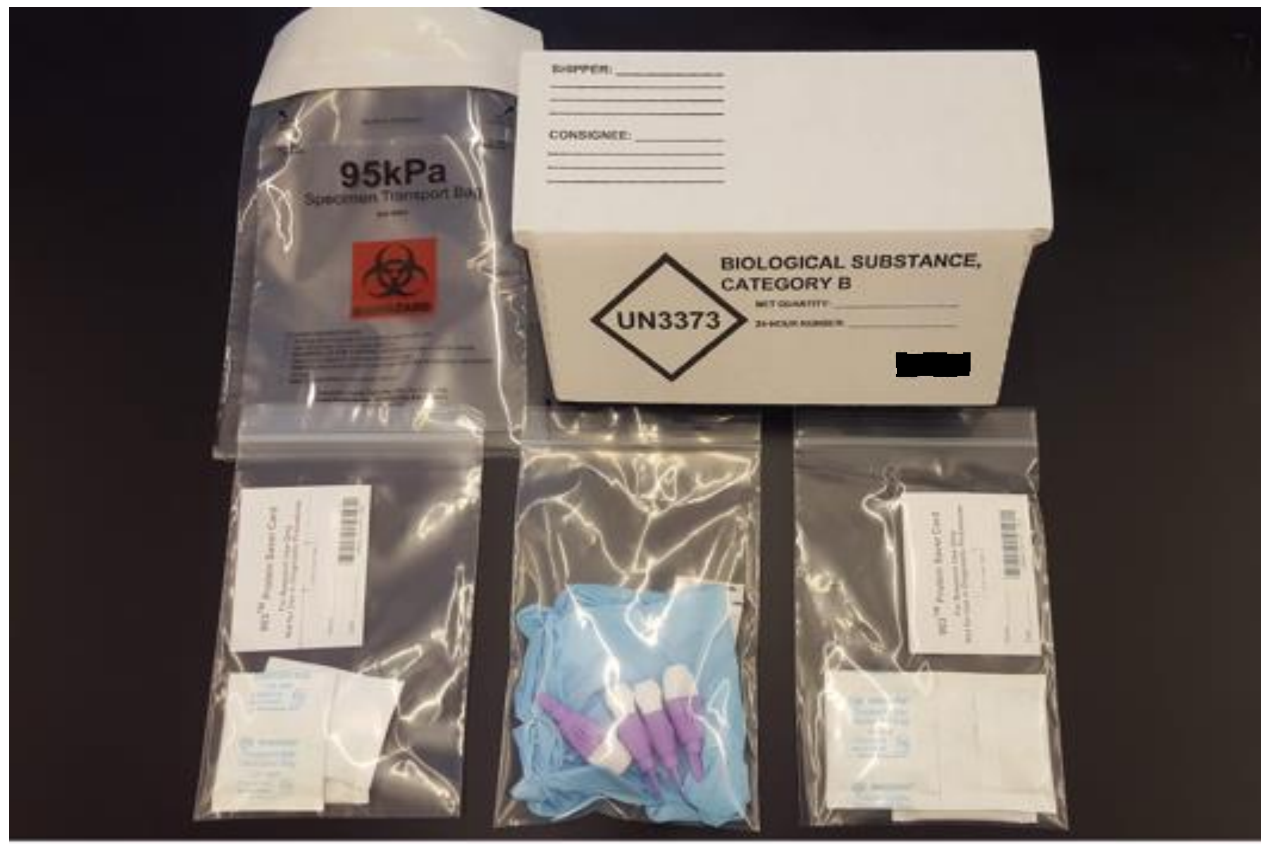

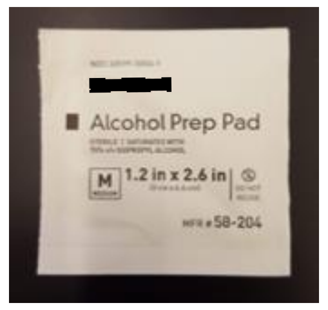

Alcohol Prep Pad (x2)

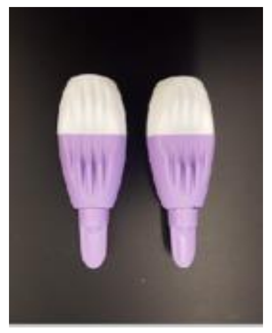

Lancets (x2)

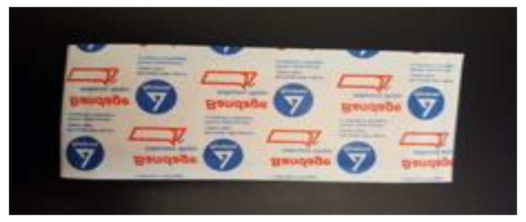

Adhesive Bandage (x2)

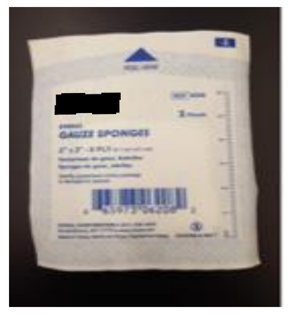

Sterile Gauze Pad (4 pads)

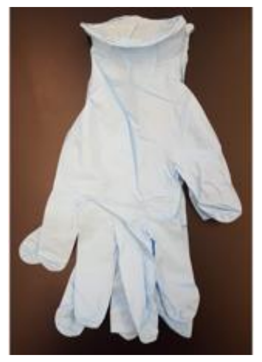

Gloves (x2)

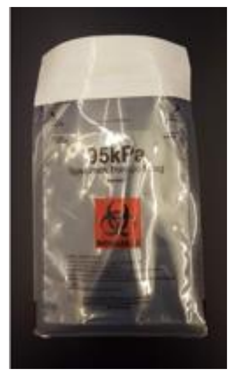

Return Bag

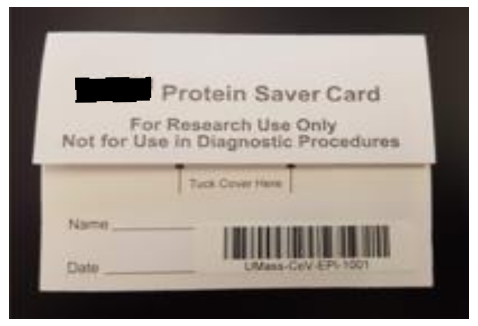

Dried Blood Spot Collection Card (1 per participant)

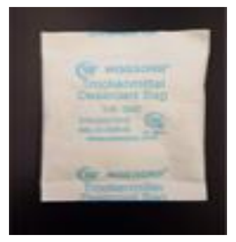

Adsorbent bags

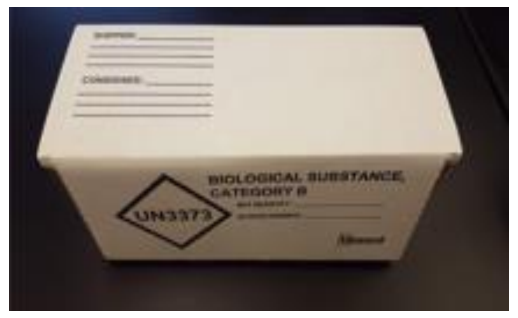

Box with Prepaid UPS Return Label and Tape

Getting Started

- Read these instructions completely before starting the dried blood spot collection.
- You can do this entire process yourself, but if you'd like someone to help you be sure they wear the gloves provided in this kit throughout.

IMPORTANT: Report Barcode Number
- Click the link in the most recent email you received to review the 4-minute instructional video and then **enter the last 4 digits of the test barcode from your blood spot card in the survey.**

Before you start
- Drink a glass of water to make sure that you are hydrated before starting the collection.
- Make sure your hands are warm to help with blood flow.

Preparing a clean surface for use
- Lay out a strip of 3 paper towels on a flat surface or table top.
- Place collection card, lancets (2), alcohol prep pad, gauze pads (2), and adhesive bandage on the paper towels.
- Place the collection card in front of you, face up with the circles exposed.
    - You do not need to write on the card. Be sure you reported your barcode number (see instructions above).
- Tear open and remove the alcohol wipe, bandage, and gauze pads, and place them on the paper towel.

1) Clean Hands and Prepare Finger

- Wash your hands with hot water and soap. This also helps to make sure your hands are very warm before starting the collection.
- Stand or sit in front of the flat surface/table top with the collection materials in front of you.
- Choose your dominant hand to use for the collection.
- Gently massage or shake your dominant hand downward for about 15 seconds to get the blood to collect in your fingers.
- Wipe the fleshy side (closer to the pinky rather than the thumb) of either your middle or ring finger of your dominant hand with the alcohol wipe.

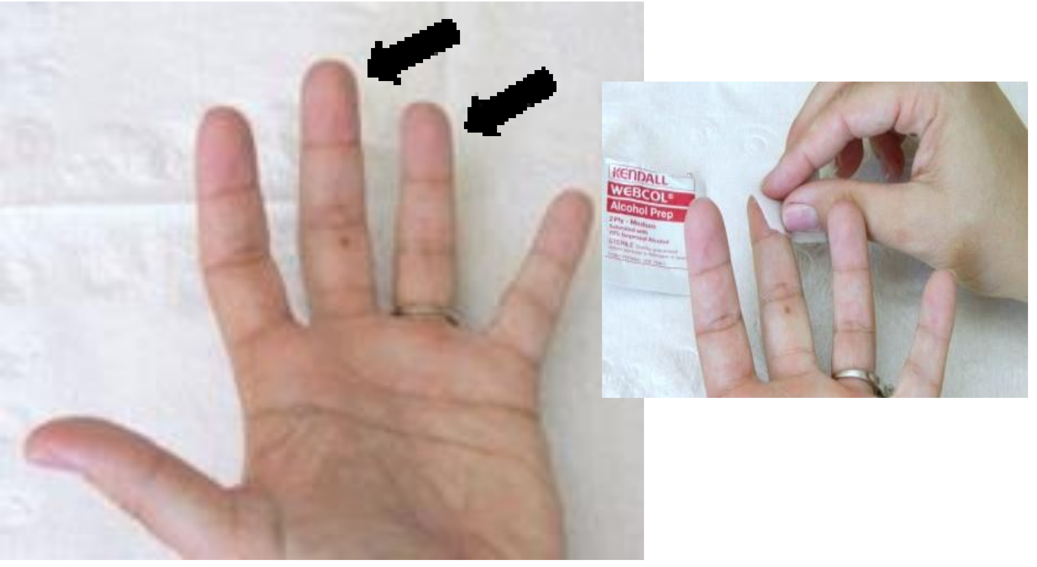

2) Remove Cap from Lancet

- Twist the cap one full turn and pull until the cap comes off.

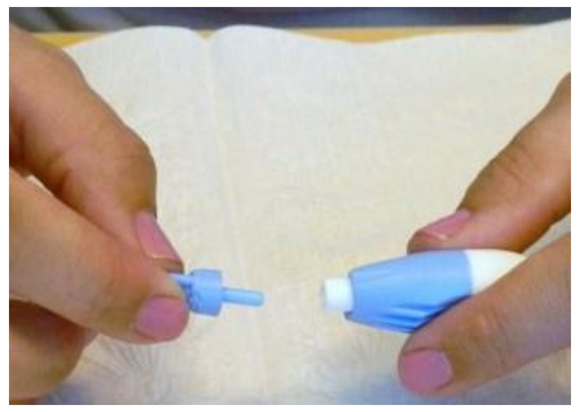

3) Performing the Finger Prick

Bring the thumb of your dominant hand up to tip of the chosen finger. Use the thumb to make the prick surface stretched tight.

- Place the lancet on the fleshy side (closer to the pinky rather than the thumb) of the chosen finger. By pricking the fleshy side facing the pinky, your hand will be in a more comfortable position to let the blood drops fall from your finger.

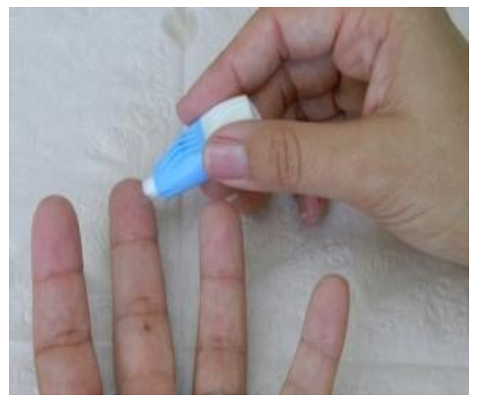

- Do **NOT** place the lancet in the middle of the finger pad or near the nail.
- Hold lancet firmly against the tight portion of the chosen finger. Press lancet down hard until it “clicks”.
- **Using one gauze pad, wipe away the first drop of blood from the pricked finger.**

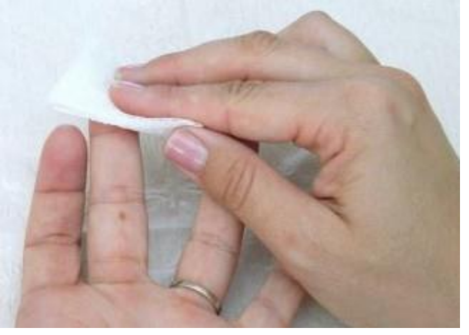

- Milk the finger to make sure blood is flowing freely. To milk your finger, place your thumb at the bottom of the chosen finger, gently press down and slide your thumb up to the finger pad.

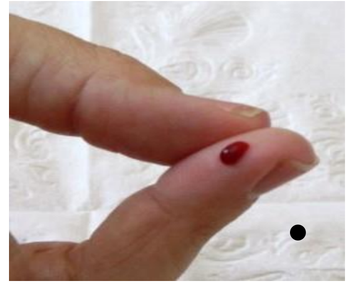

- Continue to slide your thumb up from the bottom to the finger pad.

4) Collecting the Blood Sample

- Point the pricked finger down and hold it over the center of the 1st circle on the collection card. Gently milk the finger in this position.
- Wait for one LARGE drop of blood to form. Let the blood drop fall to the center of the 1st circle on the collection card.

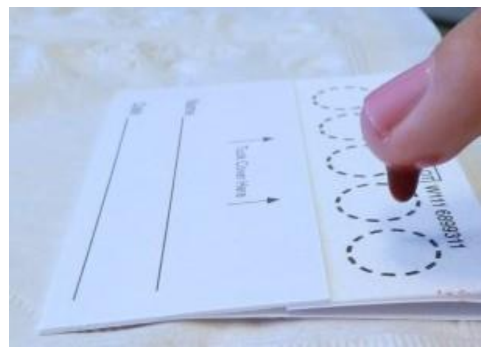

- Do **NOT** bring the paper up to catch the drop. Do **NOT** smear, blot, or touch your finger to the card.
- Do **NOT** place a second drop in a circle.
- Follow the steps above until all 5 circles have been filled with **ONE** LARGE drop of blood per circle.

YES **NO**

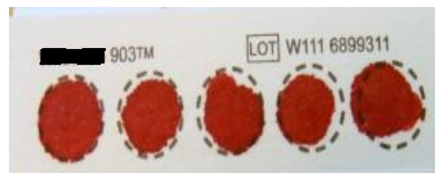

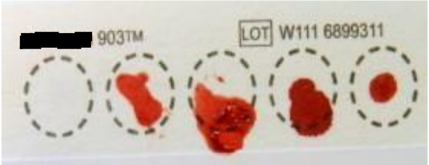

- To help with blood flow, massage the hand of your chosen finger with the other hand and milk up from the bottom of the hand to the finger prick. Also you can wipe the pricked finger with a gauze pad and start milking the finger again.
- If you are having trouble completing the collection card, prick a different finger with the 2nd lancet and follow the above steps.
- When finished, place a gauze pad on the pricked finger and apply pressure to stop any bleeding. Once bleeding has slowed, place a bandage on the pricked finger.

5) Mailing the Blood Sample

- **IMPORTANT: Allow the collection card to air dry at least 4 hours at room temperature. If possible, allow the card to dry overnight at room temperature, but protect from pets/insects and sunlight.**
- Place the blood spot collection card in the return bag with the adsorbent bags. Follow the instructions provided to close and seal the bag.

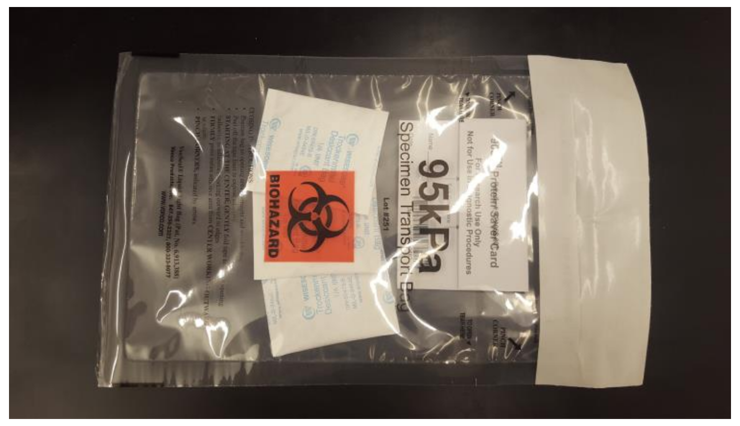

- Place the return bag inside the shipping box. Place any used supplies (lancets, alcohol pads, etc.), unused supplies, and unused blood spot card (if applicable) in ziplock bags into the box as well.
- Use the provided package tape to seal the box closed (this tape requires moistening with sponge or paper towel).
- **Mailing instructions: the box comes with a prepaid UPS label. Attach this to the box covering the old label. Then, leave with your regular mail for pickup or drop off at any US Post Office. If you prefer to schedule a pickup.** https://tools.usps.com/schedule-pickup-steps.htm

Please mail the package back to UMass by July 24.

Thank you!

- If you have requested your results, we will mail them to your address. This may take several weeks or longer.
